# Prevalence and risk factors of perinatal asphyxia in newborns in Colombia: a systematic review and meta-analysis

**DOI:** 10.3389/fped.2025.1534397

**Published:** 2025-12-04

**Authors:** Sergio Agudelo-Pérez, Valeria Mejía Echeverría, Laura Valentina Medellín Ortiz, Tania Dayana García Cárdenas, Juan Miguel Pérez Flórez, María José Maldonado

**Affiliations:** Department of Pediatrics, Universidad de La Sabana, Chía, Colombia

**Keywords:** asphyxia neonatorum, infant newborn, prevalence, meta-analysis, hypoxic-ischemic encephalopathy

## Abstract

**Background:**

Perinatal asphyxia (PA) is a critical neonatal condition and a leading cause of morbidity and mortality, particularly in low- and middle-income countries. Despite its clinical relevance, data on the prevalence and risk factors for PA in Colombia remain limited. This study aimed to estimate the cumulative prevalence of PA and identify associated risk factors in the Colombian neonatal population.

**Methods:**

A systematic review and meta-analysis were conducted in accordance with the PRISMA guidelines. A comprehensive search of PubMed, Scopus, LILACS, and Web of Science databases was performed between July and August 2024. No restrictions were applied regarding the publication year. Data were extracted, synthesized, and analyzed using random-effects models. Heterogeneity was assessed using *I*^2^ and τ^2^ statistics, and potential publication bias was evaluated using Funnel Plot analysis and Egger's test.

**Results:**

Nine studies with 16,778 patients were included. The cumulative prevalence of PA was estimated to be 5.92% (95% CI: 0.00, 25.03%) with significant heterogeneity (*I*^2^ = 99.2%). Key risk factors associated with PA included meconium-stained amniotic fluid (pooled OR 7.5; 95% CI 1.4–39.6), prolonged second stage of labor (pooled OR 15.1; 95% CI 1.4–157.6), and intrapartum fetal distress (pooled OR 11.6; 95% CI 1.2–114.4).

**Conclusion:**

The findings revealed a notable burden of PA in Colombia and highlighted critical inconsistencies in the diagnostic criteria and reporting practices across studies. These results underscore the urgent need to standardize definitions, strengthen perinatal surveillance systems, and improve obstetric and neonatal care. By identifying major evidence gaps and the most consistent risk factors, this review provides a foundation for future multicenter research and for the development of targeted evidence-based interventions to reduce preventable neonatal morbidity and mortality in Colombia.

## Introduction

Perinatal asphyxia (PA) is defined by the World Health Organization (WHO) as a failure to initiate or sustain spontaneous breathing at birth, a definition widely used in low- and middle-income countries (1). In contrast, the American College of Obstetricians and Gynecologists (ACOG) and the American Academy of Pediatrics (AAP) describe PA as a condition resulting from impaired gas exchange, leading to hypoxemia, hypercapnia, and metabolic acidosis. The ACOG/AAP Task Force recommends a diagnostic approach that combines biochemical criteria, such as an umbilical artery pH < 7.00 and a base deficit ≥12 mmol/L, with clinical indicators, including a 5-minute Apgar score ≤3, signs of neonatal encephalopathy, need for prolonged resuscitation, and evidence of multi-organ dysfunction (2).

Globally, PA accounts for approximately 24% of neonatal deaths, with its burden falling disproportionately in low- and middle-income countries, where it ranks as the fifth leading cause of neonatal mortality (3–5). The estimated incidence of PA in these settings ranges from 100 to 250 cases per 1,000 live births, whereas in high-income countries the rate is substantially lower, ranging from 5 to 10 cases per 1,000 live births (6). Among survivors, PA is a major cause of long-term neurodevelopmental impairment, including cerebral palsy, cognitive deficits, and epilepsy (4, 7, 8).

Several risk factors that contribute to the occurrence of PA have been identified. These include maternal sociodemographic conditions; obstetric complications such as preeclampsia, gestational diabetes, and chorioamnionitis; intrapartum factors such as prolonged labor, meconium-stained amniotic fluid (MSAF), and acute fetal distress; and neonatal factors including gestational age and birth weight (6, 9–12). On the other hand, gestational age plays a fundamental role not only in the risk of PA development but also in its pathophysiology. Ischemic and periventricular white matter lesions were more frequent in preterm neonates, whereas alterations in the basal ganglia were more frequent in term neonates than in term neonates (13).

Reliable epidemiological data on the prevalence of PA and its associated risk factors in low- and middle-income countries are essential to guide interventions aimed at reducing its occurrence. However, in Latin American countries, there is significant underreporting of PA cases in national statistics and a general scarcity of research data. These gaps are largely explained by socioeconomic disparities, geographical barriers, and limited access to healthcare services, resulting in incomplete understanding of the true burden of the condition (14). Although the overall incidence of PA in Latin America broadly aligns with global estimates, considerable variation exists between countries. For example, in Mexico, the reported prevalence in 2023 was 1.8 cases per 1,000 live births (15), whereas in Lima, Peru, a 2011–2012 study found that 14.1% of all neonatal deaths were attributable to PA (16).

A similar situation exists in Colombia, where the true burden of PA is likely to be underestimated because of underreporting. In addition, national statistics focus primarily on mortality data, overlooking the actual occurrence of the condition as only fatal cases are systematically recorded. For instance, a descriptive report from the Colombian National Institute of Health indicated that 12.4% of perinatal deaths in 2020 were attributed to PA, corresponding to a mortality rate of 0.7 per 1,000 live births (17). However, these figures lack a comprehensive analysis of underlying maternal and neonatal risk factors. Moreover, approximately one in four affected newborns may experience long-term disabilities, including cerebral palsy, neurodevelopmental impairment (motor, cognitive, or behavioral), and epilepsy (18). The scarcity of evidence-based data underscores a critical gap in the national and regional literature (19).

Given the limitations of administrative data, the scientific literature plays a crucial role in building an evidence-based understanding of PA in Colombia. This systematic review and meta-analysis aimed to estimate the prevalence of PA in newborns and to identify the most significantly associated risk factors within the Colombian context. This review focused on observational studies conducted in neonatal populations across Colombian healthcare institutions, comparing infants diagnosed with PA to those without the condition and examining both its frequency and determinants. This study sought to define the national epidemiological profile, highlight common determinants, and assess the methodological quality of available evidence. These findings are expected to inform clinical practice and public health strategies and guide the development of context-specific preventive and therapeutic interventions.

## Materials and methods

Systematic reviews and meta-analyses were conducted in accordance with the Preferred Reporting Items for Systematic Reviews and Meta-Analyses (PRISMA) guidelines (20). The PRISMA checklist is provided in Supplementary Table S1. This systematic review was submitted to the PROSPERO registry but declined (“Rejected by PROSPERO”) because the topic did not meet the registry's eligibility criteria. All stages of protocol development, study selection, and data extraction were completed before conducting the analyses to ensure methodological transparency and minimize bias.

### Search and identification of studies

A comprehensive literature search was conducted between July and August 2024 across the PubMed, Scopus, LILACS, and Web of Science (WoS) electronic databases. No restrictions were applied regarding the publication year, and studies published up to August 2024 were considered eligible. Additional searches were conducted using Google Scholar and other grey literature sources. Snowball sampling was used to identify additional relevant studies. No language restrictions were applied.

A combination of keywords and indexed terms (MeSH and DeCS) were used to develop the search strategy. Terms included *neonate*, *newborn*, *infant*, *prevalence*, *incidence*, *magnitude*, *risk factors*, *determinants*, *predictors*, *correlates*, *contributing factors*, *Colombia*, *asphyxia*, *perinatal asphyxia*, *hypoxic-ischemic encephalopathy*, *birth asphyxia*, *asphyxia neonatorum* and *suffocation*. The search strategy used for PubMed was adapted to other databases. ((((((((*N*ewborn) OR (neonate)) OR (Infant)) AND (((((((Prevalence) OR (Incidence)) OR (Magnitude)) OR (Risk factors)) OR (Determinants)) OR (Correlates)) OR (Contributing factors)))))))) ()))) AND((((((((((((Asphyxia)) OR (Hypoxic-ischemic encephalopathy)))) OR (Perinatal asphyxia))) OR (Birth asphyxia))) OR (Asphyxia neonatorum))) OR (suffocation))). The details of the search protocols used for each database and Google Scholar are listed in Supplementary Table S2.

### Eligibility criteria

#### Inclusion criteria

- Original studies conducted in healthcare institutions within Colombia.
- Observational design, including cohort, cross-sectional, and case–control studies.
- Studies evaluating maternal populations during delivery and/or neonates at birth.
- Articles reporting the prevalence of PA or at least one maternal, intrapartum, or neonatal risk factor were associated with the condition.

#### Exclusion criteria

- Narrative, systematic, or exploratory reviews.
- Conference, abstracts, posters, correspondence to the editor, and *preprints*.
- Studies using qualitative methodologies.
- Studies involving populations with congenital malformations, chromosomal abnormalities, or metabolic disorders.

### Definitions

In this review, PA was defined based on the diagnostic criteria reported in each primary study. The reviewers did not impose a uniform operational definition; instead, all studies that explicitly identified PA as a diagnosis or study outcome were eligible for inclusion regardless of the specific criteria used.

For descriptive purposes, the definitions employed in the included studies were extracted and compared against the standards proposed by the ACOG/AAP, which include biochemical evidence of metabolic acidosis in the umbilical cord or early neonatal blood gases (pH < 7.0 or base deficit ≥12 mmol/L), an Apgar score ≤3 at 5 min, evidence of moderate or severe neonatal encephalopathy, and multi-organ dysfunction.

When available, the severity of PA was recorded as mild, moderate, or severe to enable a more detailed comparison of risk factors. This approach allowed the inclusion of studies using diverse diagnostic frameworks while ensuring that definitional variability could be systematically analyzed and discussed as a source of heterogeneity across studies.

All variables identified in the primary studies as associated with an increased likelihood of PA were considered potential risk factors. No restrictions were placed on the type of variables evaluated, allowing the inclusion of social (e.g., education and family support), economic (e.g., income level), biological (e.g., maternal age and parity), and clinical (e.g., obstetric history and perinatal and neonatal conditions) domains.

### Screening and inclusion of studies

The investigators (SAP, VM, LM, and TG) independently and blindly searched for and selected studies. The initial screening was based on titles and abstracts using the Rayyan® online tool, in which duplicate records identified from the database overlaps were removed. The results of the initial screening were compared, and discrepancies were resolved through consensus among the investigators. Articles deemed relevant were retrieved from the full text for detailed and independent review by each investigator to determine their final inclusion. Discrepancies at this stage were resolved through consensus.

### Data extraction and synthesis

A data extraction matrix was developed using Microsoft Excel. The following information was systematically extracted from each study: (a) journal name, (b) author(s), year of publication, (c) city and hospital where the study was conducted, (d) type and characteristics of the population, (e) diagnostic criteria used to define PA, (f) prevalence, total number of patients included, and number of PA cases reported, and (g) risk factors, including their corresponding OR and 95% confidence intervals (95% CI).

We also intended to segregate the studied populations by gestational age (term vs. preterm newborns) given its known relevance in the occurrence of PA. However, this variable was not consistently reported across the included studies, which limited our ability to classify or analyze the data accordingly.

The extracted data were organized into tables, grouping information according to risk factors, city, type of hospital (public or private), and diagnostic criteria for PA. The data were synthesized in alignment with the study objectives and characteristics of the populations analyzed.

### Assessment of methodological quality and risk of bias

The quality of case-control studies was assessed using the Newcastle-Ottawa Scale (NOS) (21). The methodological quality of the cohort and cross-sectional studies was evaluated using the Joanna Briggs Institute (JBI) critical appraisal tool (22). Two investigators independently conducted quality assessments. Disagreements were resolved by consensus or, if necessary, by involving a third evaluator.

### Statistical analysis

Meta-analyses were performed to estimate the prevalence of PA and its associated risk factors. All analyses were performed using RStudio (version 2025.09.1; Posit Software, PBC) and the meta package. Metaprop and metagen functions were used in the meta-analysis of the prevalence and risk factors, respectively. A random-effects model with Hartung–Knapp adjustment was used to account for the anticipated heterogeneity.

### Meta-analysis of prevalence

Observational studies reporting data from healthcare institutions in Colombia were included to estimate pooled PA prevalence. The Prevalence and corresponding 95% CI were calculated using a random-effects model with Hartung–Knapp correction. Statistical heterogeneity was evaluated using the *I*^2^ and τ^2^ statistics. Given the anticipated heterogeneity in the diagnostic definitions of PA across studies, a sensitivity analysis was performed to assess the robustness of the pooled prevalence estimates by excluding studies with unspecified or non-standard diagnostic criteria.

### Meta-analysis of risk factors

For the analysis of risk factors, only the variables reported in more than one study were included to allow quantitative synthesis. The main factors analyzed were acute fetal distress, MSAF, prolonged second stage of labor, preeclampsia, and maternal morbidities. The effect size for each was expressed as OR with 95% CIs, estimated using a random-effects model with Hartung–Knapp correction.

When studies reported alternative effect measures such as relative risks (RR) or prevalence ratios (PR), they were converted to log odds ratios (logOR) to ensure comparability. Heterogeneity was again assessed using the *I*^2^ and τ^2^ statistics. Although a sensitivity analysis excluding individual studies was planned to evaluate their influence on the pooled results, the small number of eligible studies did not allow this approach. The risk factors reported in only one study were qualitatively summarized to provide a comprehensive overview.

### Assessment of publication bias

Potential publication bias was explored using a funnel plot and Egger's test to assess asymmetry in the distribution of effect estimates relative to their standard errors. However, given the limited number of included studies, these methods should be interpreted with caution.

## Results

**Figure 1:**
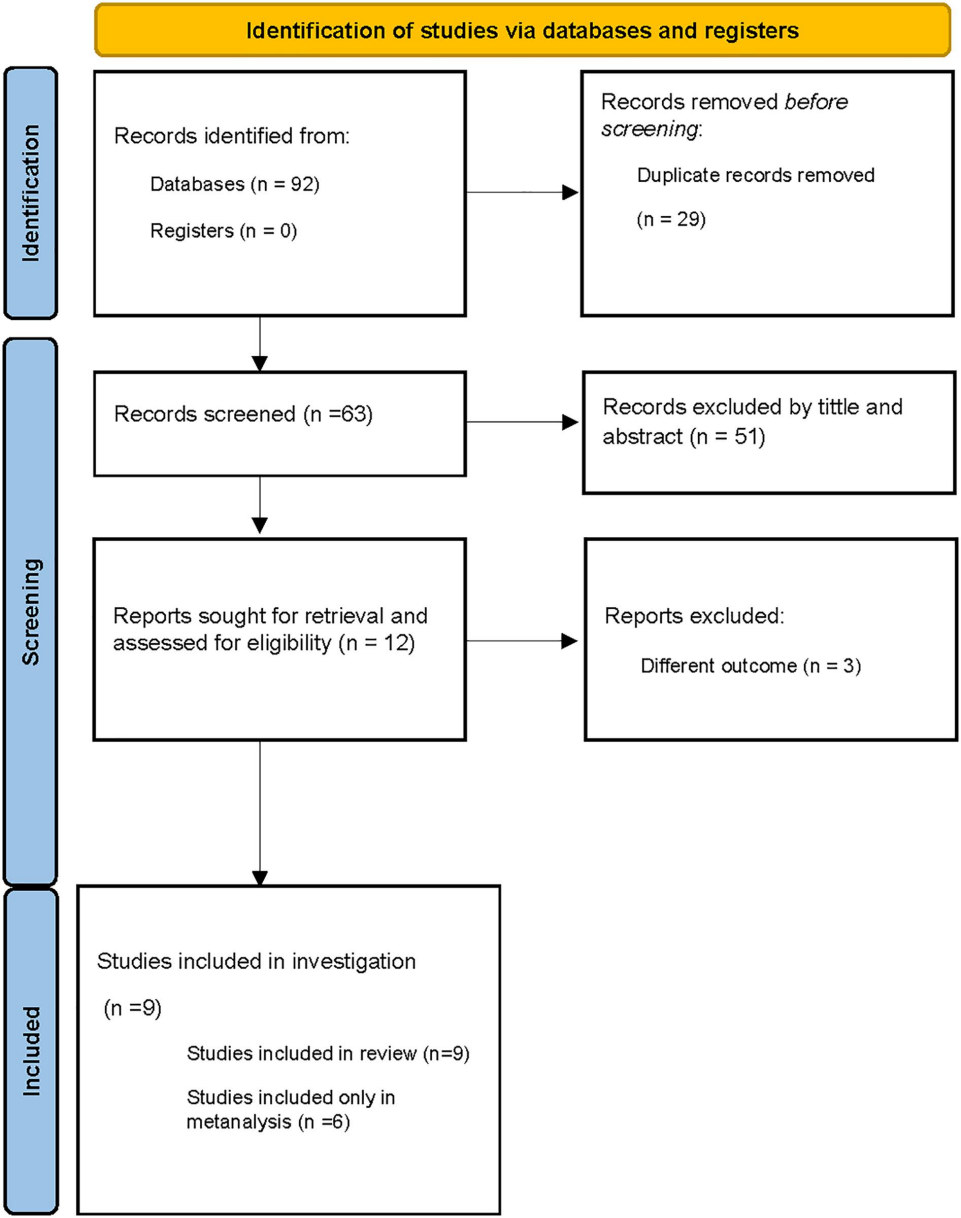
Diagram PRISMA of information flow through phases of systematic review.

A total of 92 records were identified through the database searches. After removing 29 duplicates, 63 records were screened on the basis of their titles and abstracts. Of these, 51 were excluded as they did not meet the inclusion criteria. The remaining 12 full-text articles were assessed for eligibility, and 3 were further excluded due to unsuitable methodology, non-Colombian setting, or lack of relevance to PA. Ultimately, nine studies were included in the systematic review, of which six were eligible for the meta-analysis (Figure 1). The primary reasons for exclusion were unsuitable methodological design, studies conducted outside the Colombian context, and articles that did not address the research topic.

Four articles were selected for the meta-analysis of PA prevalence (23–26). For the meta-analysis of risk factors, four studies were included for acute fetal distress (24, 27–29), four for preeclampsia (24, 27–29), three for maternal morbidity (9, 26, 29, 30), four for MSAF (27–30), and three for the prolonged expulsive phase (27, 28, 30). Regarding the study design, two studies included in the prevalence analysis were cross-sectional, whereas the other two were cohort studies. Five studies used a case-control design to analyze risk factors.

The characteristics of the included studies are summarized in Table 1. All the included studies were conducted in major cities and departmental capital in Colombia. Three studies were conducted in Bogotá, three in Cali, one in Barranquilla, one in Cúcuta, and one in Popayan. These cities were the most populous in terms of their departments. Regarding hospital type, six studies were conducted in public hospitals, whereas three were performed in private institutions. All the studies were conducted in high-complexity university hospitals. With respect to population characteristics, four studies reported gestational age (23, 24, 28, 29).

**Table 1:** Characteristics of the studies included in the review.

Author, year	City	Study design	Hospital type	Diagnostic criteria	Population characteristics	Risk factors
Sánchez et al. (9)	Popayán	Matched Case-control study	Public, high-complexity, university hospital (neonatal intensive care unit—NICU-: 12 intensive/intermediate care beds; 45 neonatal cribs).	Medical diagnosis of perinatal asphyxia or hypoxic-ischemic encephalopathy (HIE) according to institutional clinical guidelines, based on the national guidelines issued by the Colombian Ministry of Health.	*n* = 137 cases and 277 controls. Cases: Newborns >37 weeks of gestational age, with symptom onset within the first 24 h of life.	Ethnicity; extreme maternal age; marital status; place of residence; socioeconomic stratum; health insurance status; obstetric history; gestational age; mode of delivery; adequacy of prenatal care; maternal complications during the third trimester; and male sex.
Moncada et al. (23)	Cúcuta	Cross-sectional	Public, high-complexity university hospital (NICU: 14 beds, Neonatology ward: 20 beds).	Not specified; diagnosis based on medical record coding for perinatal asphyxia.	*n* = 58 (out of 6,709 live births in 2021) Newborns without congenital malformations. Female: 47% and Male: 53%. Term: 81%, preterm 17% and post-term: 2%.	Ethnicity; maternal age >40 years; educational level; health insurance status; birth weight; maternal obesity (BMI >30); insufficient prenatal care; previous cesarean section; multiparity; multiple pregnancy; male sex; late/extreme prematurity; cesarean delivery; induced labor; uterine tachysystole; oligohydramnios/polyhydramnios
Rincón et al. (27)	Bogotá	Matched case-control (1:5)	Private, high-complexity university hospital (NICU: 15 beds, Neonatology ward: 13 beds, including intermediate and basic neonatal care services).	Case definition followed AAP/ACOG guidance; diagnosis was made if ≥1 of the following was present: (1) metabolic acidosis in umbilical arterial blood at birth (pH < 7.0 and base deficit ≥12 mmol/L), (2) Apgar 0–3 at 5 min, or (3) neurologic impairment consistent with hypoxic-ischemic injury with multi-organ dysfunction.	*n* = 306 (51 cases and 255 controls). Newborns without congenital malformations.	Maternal age <18 or >35 years; unmarried status; positive maternal medical history; primigravidity;≤3 prenatal visits; abruptio placentae; hypertensive disorders of pregnancy; third-trimester hemorrhage; prolonged ROM (>24 h); oligohydramnios; maternal UTI; vaginal candidiasis; fetal tachycardia; abnormal intrapartum monitoring; prolonged/arrested labor; failure of dilation/progression; second stage ≥1 h; male sex; GA ≤36 weeks (Ballard); fetal dystocia; instrumental delivery; meconium- or blood-stained amniotic fluid; nuchal cord; birth weight ≤2,500 g
					No additional demographic data reported.	
Torres et al. (26)	Cali	Prospective observational	Public, high-complexity university hospital.	Sarnat 1: hyperalert state, disinhibition of Moro reflex and stretch reflexes and sympathetic effects; Sarnat 2: obnubilation, hypotonia, strong distal flexion and multifocal convulsions Sarnat 3: with stupor or flaccidity, convulsions and suppressed brain stem and autonomic functions. Arterial gases in the first hour of life (no value reported).	*n* = 111. Newborns >35 weeks, without major congenital malformations, chromosomal abnormalities, metabolic disorders, or neuromuscular diseases.	Maternal age (<18 or >35); >7 prenatal visits; hypertensive disorders; dystocia; abnormal fetal heart tracing; maternal infections; third-trimester hemorrhage; cord pathologies; stress test abnormalities; vaginal or cesarean delivery; regional/general anesthesia; birth weight alterations (<2,500 g or >4,000 g); meconium-stained amniotic fluid
Del Riesgo-Prendes et al. (24)	Bogotá	Observational, hospital-based (descriptive analytic)	Private, high-complexity university hospital.	Diagnosis according to the American Academy of Pediatrics and the American College of Obstetricians and Gynecologists criteria. These include evidence of metabolic acidosis (umbilical arterial pH < 7.0 and base deficit ≥ 12 mmol/L), Apgar score 0–3 at 5 min, and neurological signs of hypoxic-ischemic encephalopathy.	*n* = 124 (1.45% of 8,837) (124 newborns with perinatal asphyxia and a comparison group of 763 newborns with respiratory distress but without criteria for asphyxia).	Maternal age <18 or >35; primiparity; previous abortions; gestational diabetes; hypertensive disorders; thyroid disease; poly/oligohydramnios; placenta previa; placental insufficiency; chorioamnionitis; PROM;<6 prenatal visits or none; vaginal/abdominal delivery; fetal distress; SGA; preterm birth; male sex; hypoglycemia; sepsis; neurological signs (hypotonia/hypoactivity)
			Neonatal Intensive (NICU: 15 beds		Inclusion: preterm and term infants without congenital or metabolic malformations admitted to the NICU.	
			Neonatology ward: 13 beds, including intermediate and basic neonatal care services).		Term: 47%, preterm: 52%, post-term: 0.8%.	
Pérez et al. (28)	Barranquilla	Matched case–control study (1:2 ratio)	Public, high-complexity university hospital.	Diagnosis of perinatal asphyxia based on American Academy of Pediatrics criteria:	*n* = 120 (40 cases and 80 controls). Newborns ≥26 weeks of gestational age and birth weight ≥1,000 g.	Antepartum: maternal anemia; threatened preterm labor; third-trimester hemorrhage; UTI; preeclampsia; eclampsia. Intrapartum: acute fetal distress; prolonged labor; meconium-stained amniotic fluid. Feto-neonatal: GA <37 weeks; birth weight <2,500 g. No association: maternal age, parity, mode of delivery, PROM
				Umbilical cord blood gas with pH ≤ 7.0 Apgar score 0–3 for more than 5 min Clinical evidence of hypoxic–ischemic encephalopathy (Sarnat) Biochemical evidence of multi-organ dysfunction	Cases: newborns admitted to the NICU with diagnosis of perinatal asphyxia.	
					Controls: newborns born in the same institution without criteria for perinatal asphyxia.	
					Exclusion: major congenital malformations or respiratory tract malformations	
Torres et al. (29)	Cali	Matched case–control study (1:3)	Public, high-complexity university hospital.	Diagnosis based on ACOG and AAP criteria, including:	*n* = 216 (54 cases, 162 controls).	Biological: meconium-stained amniotic fluid; chorioamnionitis; preeclampsia (risk ↑). Protective: induction of labor; fetal heart rate monitoring. Psychosocial: secondary education and ≥4 prenatal visits (risk ↓); poor social/ instrumental support (risk ↑)
				Gestational age ≥36 weeks.	Newborns ≥36 weeks of gestational age, without major congenital malformations or other explicable neurological conditions.	
				Neurological signs (convulsions, coma, hypotonia) are not attributable to other causes.		
				Arterial pH ≤7.0 or base deficit ≥12 mmol/L in the first hour of life.		
				Apgar score 0–3 at 5 min.		
				Multiorgan compromise (≥2 systems).		
				Need for advanced neonatal resuscitation.		
				Presence of sentinel events (placental abruption, uterine rupture, umbilical cord prolapse, etc.)		
Torres et al. (30)	Cali	Matched case-control study (1:3)	Public, high-complexity university hospital.	Cases were newborns ≥36 weeks' gestational age.	*n* = 224 (56 cases, 168 controls).	Premature placental abruption; prolonged expulsive phase; no oxytocin use; no partograph; maternal morbidity; meconium-stained amniotic fluid; cesarean delivery; delivery at another institution; primiparity; no drug use during labor; no partner.
				Diagnosed with moderate or severe perinatal asphyxia, who required advanced resuscitation and presented ≥1 of the following: early neurological signs (e.g., seizures, coma, hypotonia), multi-organ dysfunction, or a sentinel event compromising life. Diagnosis based on clinical criteria; umbilical arterial pH was not available for all cases	Cases: newborns ≥36 weeks admitted to the neonatal ICU with moderate/severe perinatal asphyxia.	
					Controls: newborns without asphyxia, born within one week of the case, with comparable gestational age.	
					Exclusion: major congenital malformations or syndromes	
Manotas et al. (25)	Bogotá	Cohort	Private, tertiary referral hospital.	Perinatal asphyxia defined according to AAP criteria:	*n* = 64.	Associated with mortality: initiation of TH >6 h; metabolic, hepatic, cardiac, renal and hematologic abnormalities (glycemia, platelets, enzymes, pH/base excess, lactate, troponin, creatinine). Protective: initiation of therapeutic hypothermia within 6 h.
					Inclusion criteria: neonates ≥37 weeks, ≥2,300 g, moderate/severe HIE.	
				Severe asphyxia: metabolic or mixed acidemia with cord pH < 7.0, Apgar <3 at 1 min, Apgar <5 at 5 min, neurological sequelae (seizures, hypotonia, coma), and evidence of multi-organ involvement (renal, pulmonary, hepatic, cardiac, or intestinal).		
					Exclusion criteria: major malformations, congenital heart disease, or preterm <37 weeks.	
					Female: 47% and male: 53%.	
				Moderate asphyxia: pH < 7.2 in venous cord blood, Apgar 4–6 at 1 min or < 7 at 5 min, and neurological sequelae or multi-organ compromise.	78% severe mecon, 22% moderate	

### Quality assessment and risk of bias

**Figure 2:**
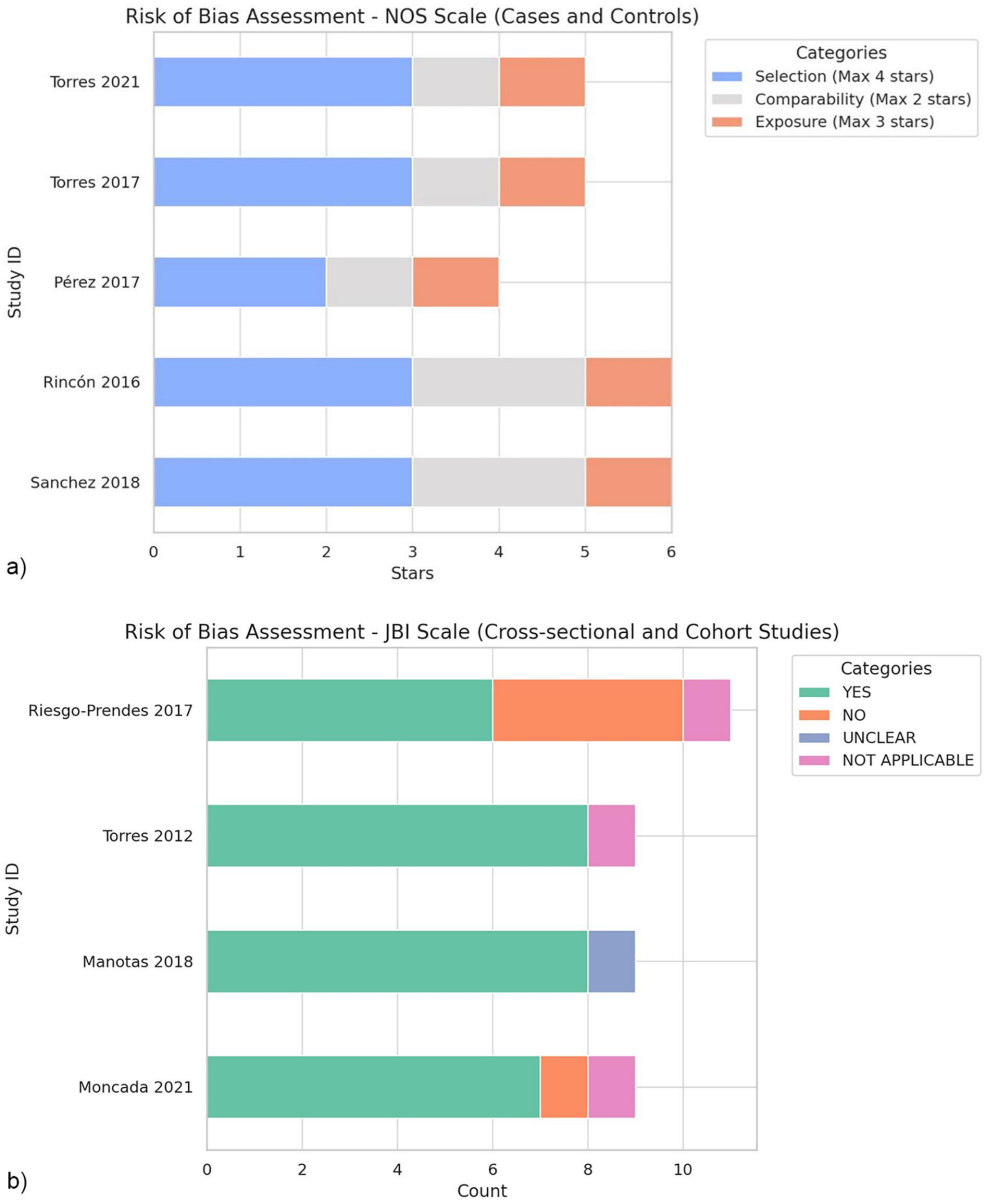
**(a)** Evaluation of the risk of bias using the New Castle-Otawa scale. **(b)** Evaluation of the risk of bias using the Joanna Briggs Institute (JBI) scale.

The quality assessment of the included studies, conducted using the NOS for case-control studies (Figure 2a) and the JBI scale for cross-sectional and cohort studies (Figure 2b), demonstrated moderate to good overall quality with a generally low risk of bias.

NOS evaluation revealed that the study by Sanchez et al. (9) and Rincón, et al. (27) achieved the highest scores, each receiving six stars. These studies demonstrated robust quality across all three categories (selection, comparability, and exposure), indicating a low probability of bias. The studies by Torres et al. (26) and Torres-Muñoz et al. (29), respectively received five stars, reflecting slight limitations in comparability and exposure categories. Pérez et al. (28), with four stars, exhibited limitations in the selection and comparability domains, suggesting a relatively higher risk of bias than other case-control studies.

The evaluation of cross-sectional and cohort studies using the JBI scale indicated moderate-to-good quality, although specific weaknesses were noted. For instance, Riesgo-Prendes et al. (24) exhibited deficiencies in the outcome domain, and several areas marked as “Not applicable,” reflecting limitations in follow-up, which is a common challenge in retrospective studies. Similarly, Moncada, et al. (23) showed limitations in terms of comparability and exposure measurements.

Overall, case-control studies demonstrated good quality, particularly in the NOS categories of selection, comparability, and exposure. In contrast, cross-sectional and cohort studies of moderate to good quality exhibited limitations primarily related to the outcome domain, likely influenced by their retrospective designs.

### Perinatal asphyxia

#### Diagnostic criteria and variability among included studies

Considerable heterogeneity was observed in the criteria used to define PA across the nine studies included (Table 1). Two studies [Sánchez et al. (9), and Moncada et al. (23)] did not specify the diagnostic criteria used (9, 23). Most of the remaining studies applied composite definitions that combined biochemical, clinical, and neurological parameters, although the thresholds and components varied.

Commonly used criteria include metabolic acidosis in umbilical cord blood gases, low Apgar scores at 5 min, and clinical evidence of hypoxic-ischemic encephalopathy or multiorgan dysfunction (Rincón et al. (27); Del Riesgo-Prendes et al. (24); Pérez et al. (28); Torres et al. (29); Manotas et al. (25)).

Of the nine studies included, only four (Torres et al. (30); Del Riesgo-Prendes et al. (24); Manotas et al. (25); Moncada et al. (23)) met the methodological criteria required to estimate prevalence. These four studies were then analyzed in greater detail to evaluate the consistency of the diagnostic definitions. Among them, diagnostic criteria remain heterogeneous: one relies solely on the diagnosis of PA without specifying criteria [Moncada et al. (23)]; two combined biochemical (umbilical cord pH < 7.0 or base excess ≥12 mmol/L) and clinical indicators such as low Apgar scores and evidence of organ dysfunction [Del Riesgo-Prendes et al. (24); Manotas et al. (25)]; and one study based diagnosis on the Sarnat staging of hypoxic-ischemic encephalopathy [Torres et al. (26)].

This methodological and diagnostic variability accounts for the extreme heterogeneity observed in the pooled prevalence estimates. None of the included studies evaluated the outcomes based on the severity of PA at birth.

#### Quantitative synthesis: prevalence of perinatal asphyxia in Colombia

**Figure 3:**
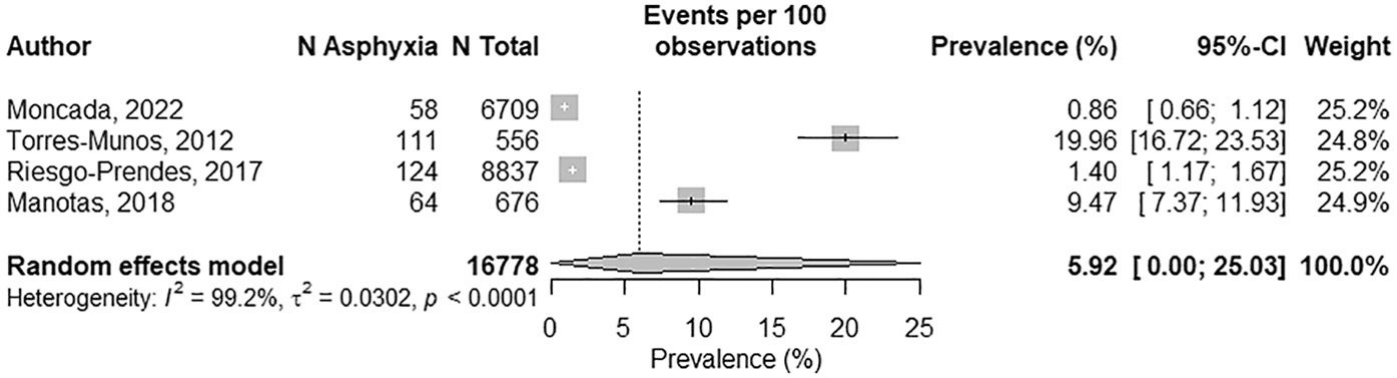
Forrest plot of the pooled prevalence of perinatal asphyxia in Colombia.

Four studies, comprising 16,778 newborns, were included in the meta-analysis (Figure 3). The pooled prevalence of PA was estimated at 5.92% (95% CI: 0.0%–25.0%). Substantial heterogeneity was observed (*I*^2^ = 99.2%, *p* < 0.001), indicating marked variability in the reported prevalence rates.

To further assess the robustness of the findings, a sensitivity analysis was performed, excluding studies that did not specify diagnostic criteria [Moncada et al. (23)]. The pooled prevalence remained low at 6.70% (95% CI 0.2%–73.4%), with persistent extreme heterogeneity (*I*^2^ = 99.6%) and 95% prediction interval of 0.0%–92.5%. This very wide interval reflects the high uncertainty and suggests that true prevalence estimates could vary substantially across future studies, mainly due to the variability in diagnostic definitions and methodological approaches rather than the influence of any single study.

**Figure 4:**
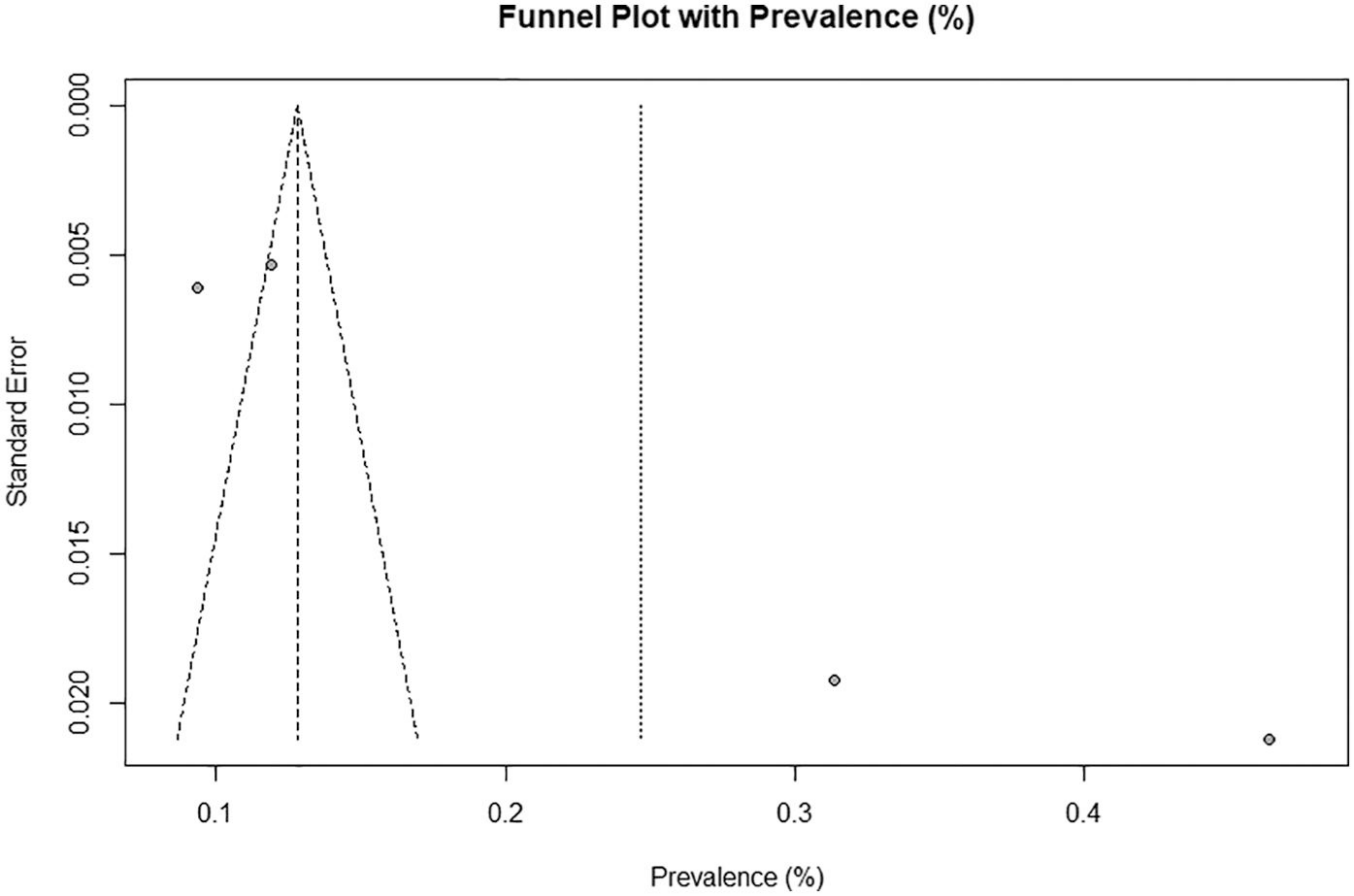
Funnel plot for publication bias.

The funnel plot (Figure 4) showed a moderately asymmetric distribution, suggesting a potential for publication bias. However, given the small number of included studies (*n* = 4), this asymmetry should be interpreted with caution as it may reflect inter-study variability rather than true publication bias. The Egger's test did not reach statistical significance (Bo = 0.161, *p* = 0.053), providing no conclusive evidence of publication bias in this dataset. Nonetheless, the limited number of studies has markedly reduced the statistical power to confidently detect biases.

Given the high heterogeneity observed, a meta-regression was initially planned to explore potential sources of variability, including risk of bias, year of publication, sample size, and diagnostic criteria. However, because of the small number of included studies (*n* = 4), this analysis was not statistically appropriate. Consequently, the ability to formally investigate determinants of heterogeneity is limited.

### Risk factors associate perinatal asphyxia in Colombia

#### Qualitative synthesis

A qualitative synthesis of the risk factors associated with PA is summarized in Table 1. Owing to heterogeneous reporting and limited data consistency across studies, many potential determinants could not be included in the quantitative synthesis and were instead described. For clarity, the identified risk factors were grouped into the maternal, antenatal, delivery, and newborn domains.

Maternal factors. Several maternal characteristics were found to be statistically associated with PA in the included studies. Rincón et al. (27), Riesgo-Prendes et al. (24), and Sánchez et al. (9) reported primiparity (OR 1.91; 95% CI 1.02–3.56) and low educational level (OR 0.32; 95% CI 0.17–0.60) as significant contributors. In addition, ethnicity emerged as a relevant determinant, with indigenous women at a higher risk than mestizo women (OR 2.07; 95% CI 1.1–3.6) (9). Torres-Muñoz et al. (29) further demonstrated that inadequate social, instrumental, or emotional support increased the likelihood of PA by 6.44-fold compared with adequate support.

Antenatal factor. Antenatal and obstetric conditions play an important role. Rincón et al. reported that a positive maternal pathological history increased the risk of PA (OR, 6.00; 95% CI, 1.55–23.19), as did primigravity (OR, 1.91; 95% CI, 1.02–3.56) (27). Similarly, Pérez rez et al. identified maternal anemia, threatened preterm labor, and a history of urinary tract infection during pregnancy as antenatal risk factors significantly associated with PA (28).

Delivery-related and Newborn Factors. Several delivery-related and neonatal factors are associated with PA. Rincón et al. (27) reported that instrumental delivery was strongly associated with PA (OR 18.8; 95% CI 3.69–39.55), as was birth weight ≤2,500 g (OR 8.88; 95% CI 3.73–21.15). In a separate study by Pérez et al. (28) found that gestational age <37 weeks (OR 10.5; 95% CI 4.37–25.3) and low birth weight (OR 4.5; 95% CI 1.83–11.1) were also significant neonatal risk factors. In addition, Torres-Muñoz et al. (30) identified cesarean delivery as a factor associated with higher odds of PA (OR 12.9; 95% CI 4.95–34.86).

#### Quantitative synthesis: risk factors associated with perinatal asphyxia within Colombia health institutions

**Figure 5:**
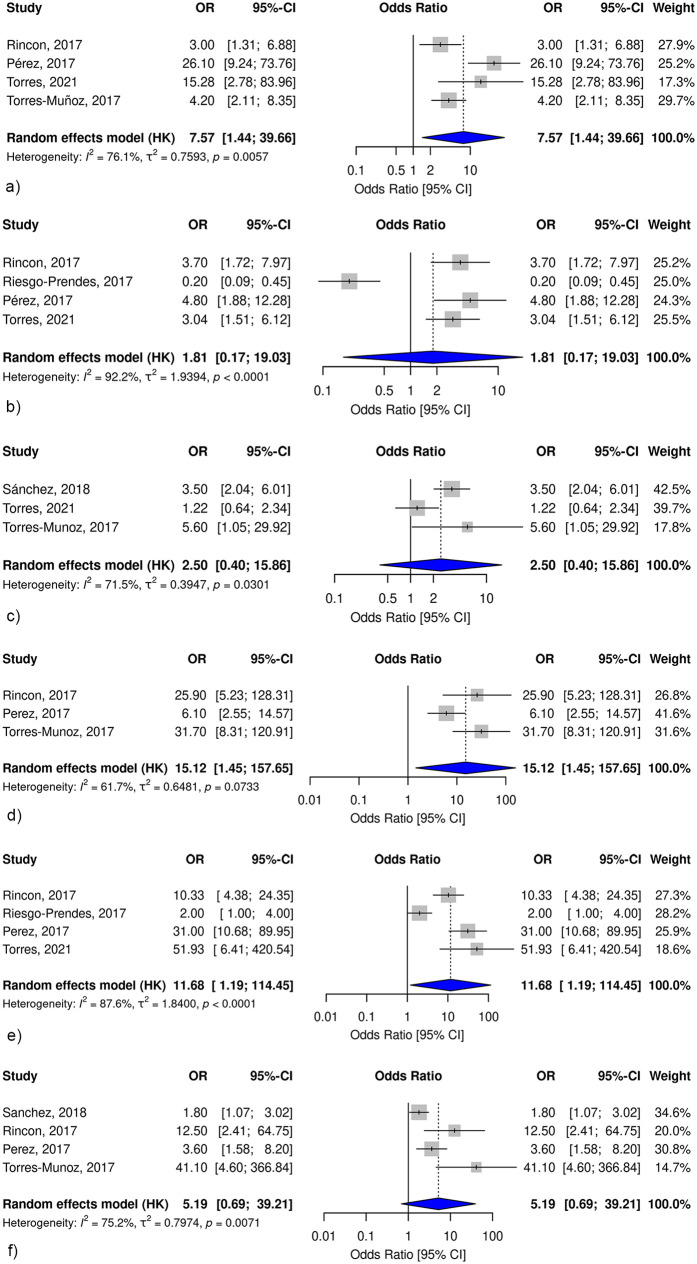
Forest plots of the pooled odds ratios for risk factors associated with perinatal asphyxia in Colombia. **(a)** Meconium-stained amniotic fluid and perinatal asphyxia. **(b)** Preeclampsia and perinatal asphyxia. **(c)** Maternal morbidity and perinatal asphyxia. **(d)** Prolonged second stage of labor and perinatal asphyxia. **(e)** Intrapartum fetal distress and perinatal asphyxia. **(f)** Third-trimester bleeding and perinatal asphyxia. Each panel presents the pooled odds ratio with 95% confidence intervals from random-effects models, including individual study estimates and corresponding weights.

Meconium-Stained Amniotic Fluid. Four studies evaluated the association between MSAF and PA (Figure 5a). A meta-analysis using a random-effects model showed that the presence of MAF fluid significantly increased the odds of PA by 7.57-fold (pooled OR, 7.57; 95% CI, 1.44–39.66). Heterogeneity among studies was moderate (*I*^2^ = 76.1%, *p* = 0.005), suggesting variability in study design and population characteristics, but consistent direction of effect.

Preeclampsia. The association between preeclampsia and PA was assessed in four studies (Figure 5b). The pooled estimate from the random-effects model did not show a statistically significant association (pooled OR, 1.81; 95% CI 0.17–19.03). However, these studies exhibited substantial heterogeneity (*I*^2^ = 92.2%, *p* < 0.0001).

Maternal Morbidity. Three studies assessed the association between maternal morbidity during pregnancy and PA (Figure 5c). The pooled analysis suggested an increased risk (pooled OR, 2.50; 95% CI 0.40–15.86), although the association was not statistically significant and the 95% CI was wide. The heterogeneity among the studies was moderate (*I*^2^ = 71.5%, *p* = 0.03), which may have contributed to the wide 95% CI and limited interpretability of the findings.

Prolonged Second Stage of Labor. The association between prolonged second stage of labor and PA was analyzed in three studies (Figure 5d). The meta-analysis revealed a significant association, with a 15.12-fold increase in odds of PA (pooled OR, 15.12; 95% CI 1.45–157.65). However, heterogeneity was considerable (*I*^2^ = 61.7%) and the wide 95% CI underscores the uncertainty surrounding the precision of this estimate.

Intrapartum Fetal Distress. Four studies examined the role of intrapartum fetal distress in PA (Figure 5e). The pooled results demonstrated a strong association, with intrapartum fetal distress increasing the odds of PA by approximately 11.68 times (pooled OR 11.68; 95% CI 1.19–114.45). Heterogeneity was considerable (*I*^2^ = 87.6%).

Third-Trimester Bleeding. The relationship between third trimester bleeding and PA was examined in four studies (Figure 5e). The Pooled analysis did not reveal a statistically significant association (pooled OR, 5.19; 95% CI 0.69–39.21). Substantial heterogeneity was observed among the studies (*I*^2^ = 75.2%, *P* = 0.007).

## Discussion

This systematic review and meta-analysis provide the first comprehensive synthesis of the prevalence and risk factors of PA in Colombia, based on institutional-level data. The pooled prevalence was 5.92% (95% CI: 0.00%–25.3%), with risk factors including MSAF, prolonged second stage of labor, and intrapartum fetal distress. While the pooled prevalence provides a general overview of the available evidence, the small number of included studies (*k* = 4), the very wide 95% CI, and the high heterogeneity (*I*^2^ = 99.2%), which is expected in low- and middle-income settings where diagnostic and reporting practices vary widely, indicate substantial statistical uncertainty. To further characterize this variability, a 95% prediction interval was calculated (0.0%–92.5%), revealing that the true prevalence could vary widely among future studies owing to the lack of standardized diagnostic and methodological approaches. Consequently, the pooled estimate should be interpreted as exploratory and descriptive rather than conclusive, primarily to highlight the evidence gap and the need for more methodologically consistent national research and multicenter studies to generate reliable national data on PA epidemiology in Colombia.

Furthermore, the variability in methodological quality among the included studies may have contributed to the observed heterogeneity. While most studies demonstrated moderate to good quality according to NOS and JBI assessments, some cross-sectional and cohort studies exhibited weaknesses in the outcome measurement and comparability domains. These limitations likely introduced variability in the prevalence estimates and reduced the precision of the pooled results, emphasizing the need for future high-quality, prospectively designed studies to improve the reliability of national estimates.

A notable limitation of the included studies was the marked heterogeneity in the diagnostic criteria for PA. Some studies used comprehensive definitions, including biochemical markers (e.g., umbilical artery pH < 7.0) and neurological assessment tools, such as the Sarnat staging system, whereas others relied primarily on clinical parameters, such as Apgar scores or the need for advanced resuscitation. This inconsistency likely contributed to the substantial between-study heterogeneity in prevalence estimates and may have affected the comparability of risk factor associations. These variations underscore the urgent need for standardized diagnostic frameworks to ensure accurate case identification and improve cross-study comparability.

The lack of standardization across Colombian epidemiological studies contrasts sharply with international recommendations, which emphasize four essential criteria: biochemical evidence of metabolic acidosis at birth, determined by umbilical cord or early neonatal blood gas analysis (pH < 7.0 or base deficit ≥12 mmol/L), clinical evidence of moderate or severe neonatal encephalopathy, neuroimaging findings, particularly magnetic resonance imaging (MRI) consistent with hypoxic-ischemic injury, and exclusion of alternative identifiable causes (2, 31). The absence of standardized protocols in Colombia limits the generalizability of our findings and complicates the formulation of evidence-based public health strategies. Therefore, future epidemiological studies aiming to establish the burden and causes of PA in Colombia should adopt standardized diagnostic criteria to improve disease characterization and generate reliable evidence to inform policies and targeted interventions. The adoption of these standardized criteria across Colombian institutions is essential for the accurate surveillance, early intervention, and policy-driven reduction of preventable neonatal deaths.

Furthermore, the limited number of epidemiological studies on PA in Colombia contrasts with the substantial clinical burden of this condition. Although global reductions in PA-related mortality have been achieved, the incidence remains high and continues to fall short of the Sustainable Development Goals for 2030 (32). In Colombia, based on national registry data, PA is the third leading cause of neonatal death, accounting for 24% of all neonatal deaths, with a mortality rate of 2.2 per 1,000 live births (33). Beyond mortality, PA imposes a significant long-term burden on the affected infants, their families, and the healthcare system.

The results of this study contrast with those of meta-analyses from other regions. For example, in Ethiopia, the pooled prevalence of PA ranges from 19.3% to 24.06% across different reviews (34–36). Similarly, a meta-analysis from Central and East Africa reported a prevalence of 15.9% (95% CI: 10.8%–21.0% (37). These figures highlight the comparatively lower prevalence reported in Colombia, which may reflect differences in healthcare access, diagnostic definitions, data collection methodologies, the denominator of live births, inclusion of NICU-only populations, and geographic coverage across studies.

However, research on PA in Latin America is limited. In Brazil, the mortality rate due to PA has been reported at 0.65 per 1,000 live births in various neonatal units (38). Similarly, the Iberoamerican Neonatology Network (SIBEN) reported that hypoxic-ischemic encephalopathy (HIE), a consequence of PA, affected 5.1% of neonates admitted to neonatal intensive care units (NICUs) with a mortality rate of 42% (14). This prevalence among Latin American NICUs belonging to the SIBEN closely aligns with the findings of the present study. Therefore, there is an urgent need for more robust and regionally representative data from Colombia and Latin America to better characterize the true burden of PA.

In contrast, the risk factors identified in this meta-analysis, MSAF, intrapartum fetal distress, and prolonged labor, are consistent with robust evidence from international meta-analyses reporting strong associations between these variables and PA (34–36). Similarly, other factors described in studies from South America and global reviews, such as emergency cesarean section, inadequate prenatal care, maternal anemia, low birth weight, labor induction, and non-cephalic presentation (14, 34–36), were confirmed as significant predictors in the qualitative synthesis. However, they could not be included in the meta-analysis because of the inconsistent reporting across the included studies.

It is noteworthy that some pooled OR, particularly for intrapartum fetal distress and prolonged second stage of labor, presented wide 95% CI, despite reaching statistical significance. This imprecision likely reflects the substantial heterogeneity observed among studies, differences in diagnostic definitions, and small sample sizes. These factors limit the strength of the observed associations and highlight the importance of conducting future studies with standardized diagnostic approaches, larger populations, and improved control of confounding variables to confirm these findings.

From a policy perspective, the identification of these risk factors suggests potential intervention points to strengthen maternal and perinatal health strategies in Colombia. Although the findings should be interpreted cautiously, they highlight the importance of reinforcing prenatal surveillance systems, ensuring timely access to obstetric and neonatal care, and promoting the use of standardized diagnostic criteria across healthcare facilities.

Strengthening targeted strategies for the management of high-risk pregnancies could facilitate the earlier recognition and prevention of complications associated with PA. Collaboration between healthcare providers and policymakers is essential to translate these preliminary findings into actionable improvements in perinatal care.

In other regions of Latin America and the Caribbean, the implementation of comprehensive maternal–neonatal health packages has been associated with up to a 23% reduction in deaths attributable to PA (39). In this context, the data from our review provides a useful starting point for guiding local research priorities and informing the design of future interventions aimed at reducing the burden of PA in Colombia.

The geographical distribution of the available studies represents another limitation. All studies were conducted in tertiary-level university-affiliated hospitals located in large Colombian cities, with no representation from rural or low-resource areas. This urban bias limits the generalizability of our findings to the broader national population. Recent national data have shown persistent and widening inequalities in infant outcomes across socioeconomic and geographic strata, with infants born in dispersed rural areas exhibiting an 8% higher risk of first-year mortality compared with those in urban centers (40). Such disparities highlight that the burden of PA may be underestimated in underserved regions where access to timely obstetric and neonatal care is limited. Future multicenter research including diverse geographic settings and health system levels is crucial to provide a more representative picture of PA in Colombia.

The initial study design intended to explore gestational age as a key factor influencing both the risk and pathophysiological profile of PA, recognizing that its causes and outcomes differ between preterm and term infants. However, only four of the included studies reported sufficient data to characterize the population by gestational age; therefore, this subgroup analysis could not be performed.

### Limitation and strengths of the present study

This study has several limitations. The high heterogeneity among the included studies limits the generalizability of the findings and precludes robust sensitivity analyses or meta-regression. Moreover, the small number of available studies reduced the statistical power to reliably assess the publication bias. Variability in diagnostic criteria across studies may also have influenced the pooled estimates.

The wide 95% CI observed for some pooled ORs, particularly for intrapartum fetal distress and prolonged second stage of labor, reflects substantial between-study heterogeneity, small sample sizes, and methodological variability. These factors reduce the precision of the estimates despite their statistical significance and underscore the need for cautious interpretation and more standardized, large-scale studies to validate these associations.

Additionally, the limited number of studies included in the prevalence meta-analysis severely restricted the statistical power of Egger's test and interpretability of the funnel plot. Given that these methods are inherently unreliable when fewer than ten studies are available, any apparent symmetry or asymmetry should not be interpreted as conclusive evidence for or against publication bias. Therefore, any inference regarding a potential publication bias remains highly tentative.

Despite these limitations, this study had several strengths. This is the first meta-analysis conducted in health institutions in Colombia to systematically synthesize evidence on the prevalence and risk factors of PA. The included studies demonstrated adequate methodological quality and low risk of bias, ensuring the reliability of the extracted data. Most importantly, this review provides a comprehensive synthesis of the existing evidence and identifies key research gaps, serving as a foundation for the design of multicenter studies using standardized diagnostic criteria and consistent reporting frameworks. These findings are expected to support future research priorities and evidence-based decision making regarding neonatal and maternal health policies.

## Conclusions

This study highlights the limited number of studies that have comprehensively addressed PA in Colombia and underscores the need for further research to better understand its prevalence, determinants, and clinical consequences. Unlike national mortality data, which provide only a partial picture, this analysis offers an initial synthesis of both the prevalence and associated risk factors.

Nevertheless, the findings must be interpreted with caution because of the small number of available studies, substantial heterogeneity, and variability in the diagnostic definitions, which should be interpreted as exploratory.

Rather than providing a definitive estimate or serving as a basis for clinical or policy decision-making, the results should be regarded as exploratory evidence that outlines current knowledge gaps and guides priorities for future research.

These findings emphasize the urgent need for standardized diagnostic criteria, multicenter data collection, and methodologically rigorous studies to generate reliable national estimates and to support evidence-based neonatal care and prevention strategies.

## Data Availability

The original contributions presented in the study are included in the article/Supplementary Material, further inquiries can be directed to the corresponding author.
